# Stable-Phase Peyronie’s Disease: Have Perilesional Injections of Hyaluronic Acid Come of Age?

**DOI:** 10.3390/clinpract16090162

**Published:** 2026-08-31

**Authors:** Tommaso Cai, Daniele Tiscione, Marco Puglisi, Daniele Mattevi, Simone Botti, Tommaso Ceccato, Truls Erik Bjerklund Johansen

**Affiliations:** 1 Urology Division, Santa Chiara Regional and Teaching Hospital, Trentino Integrated University Health System (ASUIT), 38123 Trento, Italy; daniele.tiscione@asuit.tn.it (D.T.); marco.puglisi@asuit.tn.it (M.P.); daniele.mattevi@asuit.tn.it (D.M.); simone.botti@asuit.tn.it (S.B.); tommaso.ceccato@asuit.tn.it (T.C.); 2 Interdepartmental Centre of Medical Sciences (CISMed), University of Trento, 38123 Trento, Italy; 3 Institute of Clinical Medicine, University of Oslo, 0010 Oslo, Norway; t.e.b.johansen@medisin.uio.no; 4 Department of Urology, Oslo University Hospital, 0010 Oslo, Norway; 5 Institute of Clinical Medicine, University of Aarhus, 8000 Aarhus, Denmark

**Keywords:** Peyronie’s disease, hyaluronic acid, penile curvature, quality of life

## Abstract

**Background**: Peyronie’s Disease (PD) is a complex andrological condition that severely compromises patients’ quality of life. While a novel formulation of hyaluronic acid (HA) has recently gained traction for treating the acute inflammatory phase, clinical evidence regarding its role in stable-phase (chronic) PD is lacking. We aimed to assess the therapeutic outcomes and safety profile of Perovial^®^, a specialized HA formulation, in patients with stable disease. **Methods**: A cohort of 47 patients with stable-phase PD was treated at a single andrological center between June and December 2025. The treatment protocol consisted of eight weekly injections targeted around the tunica albuginea plaque. Clinical efficacy was assessed 3 months post-treatment by comparing baseline data with the following outcome measures: change in penile curvature (Δ°), the international index of erectile function (IIEF-5) score, Peyronie’s Disease Questionnaire (PDQ) and Global Assessment of Peyronie’s Disease (GAPD). **Results**: At the 3-month follow-up visit, a statistically significant improvement in penile curvature was registered in 35 out of 47 patients (Δ° − 16) (*p* < 0.001). All patients had a significant improvement from baseline in terms of IIEF (20 vs. 24; *p* < 0.001) and in all PDQ domains. No major adverse events or complications were observed during the study period. At the GAPD questionnaire, 43 patients (91.2%) reported subjective improvement of penile curvature. **Conclusions**: Perilesional injection of Perovial^®^ seems a promising intervention in patients with stable-phase PD in terms of penile curvature reduction and patient-reported quality of life improvement.

## 1. Introduction

Peyronie’s disease (PD) represents a challenging andrological disease with a large impact on patients’ quality of life [1]. Several drugs and surgical procedures have been tested to reduce penile curvature and improve patients’ quality of life without satisfactory and lasting effects [2,3,4]. Intraplaque injections of collagenase from clostridium histolyticum are regarded as the gold standard treatment for reducing penile curvature and PD-related symptoms [5]. Unfortunately, collagenase of clostridium histolyticum is currently difficult to obtain outside of the USA. Due to limitations in the availability of collagenase of clostridium histolyticum, interest in hyaluronic acid (HA) injections in the management of PD has increased [6,7]. HA is an extracellular matrix glycosaminoglycan that is highly polarized at physiological pH and maintains hydration, turgor, plasticity, and viscosity in the connective tissue matrix [8]. Studies on the use of injectable HA have shown encouraging results, with reduction in plaque size and penile curvature, as well as improvement of overall sexual satisfaction [6,9,10]. All of these studies were conducted in patients with acute-phase PD, but recently, one retrospective study demonstrated that HA intraplaque injections may also improve penile curvature and quality of life in patients with stable-phase PD [11]. Although HA is used in everyday clinical practice in patients with stable-phase PD, study data on this type of patients are still lacking. The aim of the present study was to evaluate the clinical outcomes and safety of Perovial^®^ (IBSA, Lodi, Italy), a novel HA formulation, administered in perilesional injections without local anesthesia in patients with stable-phase PD.

## 2. Materials and Methods

### 2.1. Study Design and Patient Population

**Figure 1:**
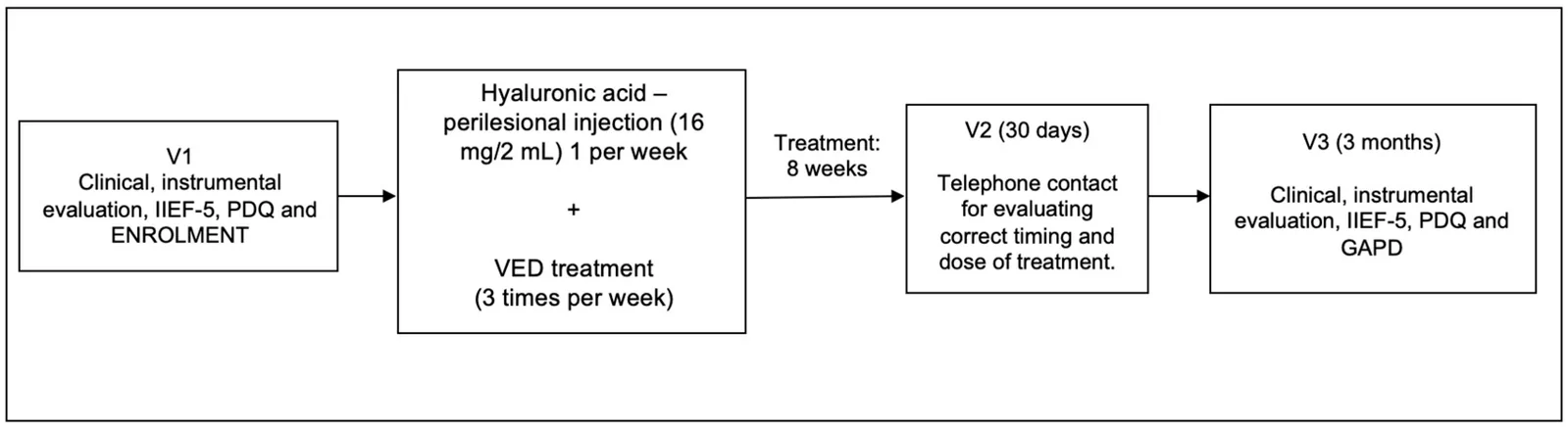
The figure shows the study schedule. IIEF-5: International Index of Erectile Function; PDQ: Peyronie’s Disease Questionnaire; GAPD: Global Assessment of Peyronie’s Disease (GAPD).

We retrospectively evaluated a cohort of adult patients (>18 years) with stable-phase PD consecutively referred to an Italian andrological referral center from June to December 2025. This study is a retrospective, exploratory analysis of a prospectively collected database (IPPdatabase; Excel-based registry developed in 2017 at home). Stable-phase PD meant duration of symptoms for more than 12 months and stable curvature for 3 to 6 months [12]. Patients should not have used penile injections of any type. They were treated according to the standardized protocol for PD at our andrological center during the study period, which included use of a vacuum device as a general recommendation (at least three times per week). All patients received one injection of Perovial^®^ at one-week intervals for 8 weeks. Injections were given in an out-patient setting without local anesthesia. Three months after cessation of therapy, patients came for a follow-up examination to evaluate treatment outcomes. Figure 1 shows the study schedule.

This study did not include a placebo group for the following reasons: (1) impossibility of obtaining collagenase of clostridium histolyticum; (2) superiority of HA intraplaque injections to the other comparators. This study is also an investigative study evaluating the feasibility of HA peri-plaque injection in patients with stable-phase PD. The lack of a placebo group has been considered in the discussion of findings.

### 2.2. Pre-Treatment Assessment

Pre-treatment evaluation consisted of detailed medical history, physical examination, and completion of questionnaires. Penile length was measured during relaxation and erection. Penile Doppler ultrasound and assessment of the angle and direction of penile curvature were made during prostaglandin-induced erection with 10 mcg alprostadil in line with the recommendations of European Association of Urology guidelines [13]. The Doppler examination also served to identify the site of maximal curvature and to exclude the presence of severely calcified plaques, which were regarded as an exclusion criterion. Self-made photography of erect penis was collected. Patients were instructed to use a fixed camera distance, controlled lighting, and camera position as part of a strict and standardized photography technique designed to reduce patient-dependent variance.

### 2.3. Questionnaires

To assess erectile function and evaluate the overall impact of the disease, all patients completed routine questionnaires which included the self-administered Italian version of International Index of Erectile Function (IIEF) [14,15], Peyronie’s Disease Questionnaire (PDQ) [16], and Global Assessment of Peyronie’s Disease (GAPD) (http://www.auxilium.com/PDQ; accessed on 23 May 2026) [17]. Pain during the injections was measured by self-assessment on a visual analogue scale (VAS) ranging from 0 to 10.

### 2.4. Treatment and Follow-Up

All patients followed our standard protocol. After inclusion, they got a penile injection with deposition of Perovial^®^ (0.8% highly purified sodium salt HA 16 mg/2 mL; IBSA, Lodi, Italy) around the tunica albuginea plaque to maximize drug distribution. During the 8-week treatment period, patients were instructed to use vacuum device therapy according to our in-house protocol by using the same device from MEDIS (Medical Service, Rozzano, Milan, Italy) during intercourse. Patients were, however, asked to avoid sexual intercourse and vacuum device therapy for 24 h after the procedure. All procedures were performed by three urologists experienced in andrological disease (T.C., D.T., M.P.). Two follow-up visits were scheduled at 30 days and 3 months after the end of the therapy, respectively. At the 30-day visit, we recorded short-term complications, checked adherence to the protocol and counseled patients on future therapy and follow-up visits. At the 3-month follow-up visit, all patients again underwent urological examination, measurement of penile length and evaluation of any residual curvature (Δ°) or penile deformity using self-made photography of the erect penis. Moreover, all patients completed IIEF-5, PDQ and GAPD scores. Before taking home photos, all patients participated in a standardized, thorough in-office training session to reduce measurement bias during follow-up. Patients were asked to use two rigorous orthogonal perspectives (top-down and left-right) with the camera held parallel to the penile shaft at a predetermined distance in order to take pictures exclusively during maximum spontaneous erection. To guarantee consistent analysis, the same operating urologist (D.T.) used conventional digital goniometry to generate all follow-up photographic measurements. The difference between baseline and follow-up in terms of penile curvature (Δ°) was calculated by using self-made photography of the erect penis.

#### Description of the Injection Technique

The treatment area was prepared using a chlorhexidine gluconate solution and conventional aseptic procedures prior to the procedure. The urologist used careful manual palpation to precisely locate the stable-phase tunica albuginea plaque; contemporaneous penile ultrasonography guidance was not used. The penis was physically stabilized when the fibrous lesion’s boundaries were established. A single pre-filled syringe of Perovial^®^ was used in the clinical protocol, and it was delivered using a 27-gauge needle that was supplied by the manufacturer. The full 2 mL amount was administered via a single-puncture needle site to maximize patient comfort and reduce tissue injury. The needle was inserted close to the edge of the lesion. The agent was dispersed circumferentially throughout the tunica albuginea lesion’s periphery. In order to promote tissue remodeling at the interface between plaque and healthy tissue, this particular deposition technique was chosen to optimize the radial distribution and uniform diffusion of the low-molecular-weight hyaluronic acid into the surrounding extracellular matrix.

### 2.5. Outcomes

The primary outcome measures were the change in penile curvature (Δ°) from baseline to the end of therapy and improvements in the IIEF-5, PDQ and GAPD scores. Secondary outcomes were pain during the procedure as assessed using the VAS score, change in penile length and residual or recurrent curvature.

### 2.6. Statistical and Ethical Considerations

Our study was conducted in line with Good Clinical Practice guidelines and the ethical principles laid down in the latest version of the Declaration of Helsinki. All anamnestic, clinical and laboratory data containing sensitive information about patients were de-identified in order to ensure analysis of anonymous data only. Non-medical staff members performed the de-identification process by means of dedicated open source software (ARX Data Anonymization Tool) [18]. Demographic and baseline characteristics have been presented as numbers and percentages. Numeric variables have been shown as mean ± standard deviation when normally distributed, and as median and interquartile range in case of non-normal distribution. McNemar’s test was used to compare categorical variables. Changes in plaque size, penile curvature, and IIEF-5, PDQ and GAPD scores from baseline to the end of treatment were assessed and analyzed using the Wilcoxon signed-rank test. Two-tailed *p* values less than 0.05 were considered statistically significant. All statistical analyses were performed using SPSS 22 for Apple Macintosh (SPSS, Inc., Chicago, IL, USA).

## 3. Results

From a total cohort of 58 patients treated from June to December 2025, data from 47 patients were selected for analysis (median age: 58 years; interquartile range (IQR); 48–69). Eleven patients were excluded due to lack of data. When comparing baseline demographic and clinical characteristics—such as age, disease features, and initial severity—no statistically significant differences emerged between the excluded subgroup and the final analytical cohort.

### 3.1. Clinical Findings at Enrolment

All patients had stable-phase PD, at least for the preceding 12 months. Dorsal curvature was the most frequent site of curvature (39 out of 47 patients, 82.9%). The median curvature was 45° (ranging from 30 to 60°). Table 1 shows all individual patient characteristics.

### 3.2. Outcomes

At the 30-day follow-up visit, all patients reported complete adherence to the protocol, including a high capability and compliance with the vacuum therapy protocol. Only one patient (2%) reported a mild pain (VAS 1–2) during the first injection, but this disappeared with the later injections. No significant adverse effects were reported. Only two patients (4%) reported a mild ecchymosis at the injection-site that disappeared spontaneously without any treatment. At the 3-month follow-up visit, a statistically significant improvement in penile curvature was registered in 35 out of 47 patients (Δ° −16) (*p* < 0.001). Twelve patients reported a reduction in penile curvature of Δ° −10. All patients experienced a significant improvement from baseline in terms of IIEF (20 vs. 24; *p* < 0.001). A statistically significant median improvement (±IQR) in all domains of the PDQ was found: physical and psychological domain −2.8 ± 3.8 (*p* < 0.001); penile pain −1.5 ± 1.9 (*p* < 0.001); symptom bother −1.3 ± 1.5 (*p* = 0.002). Regarding the GAPD questionnaire, 43 patients (91.2%) reported subjective improvement in penile curvature. Among those, eight patients (18.6%) reported a “small but important” improvement, 12 patients (27.9%) reported a “moderate” improvement, and 23 (53.5%) patients reported a “huge” improvement. Table 2 shows all clinical and questionnaires results at the 3-month follow-up visit.

## 4. Discussion

### 4.1. Main Findings

We demonstrated that perilesional injection of Perovial^®^ without local anesthesia seems a safe and effective intervention for patients with stable PD. Our eight-week protocol achieved significant reduction in penile curvature and improvement in patient-reported quality of life outcomes. At 3-month follow-up, 43 out of 47 patients (91.2%) reported significant improvement at GAPD questionnaire, and 35 out of 47 patients (74%) reported penile curvature improvement (Δ° −16) (*p* < 0.001). No major adverse events or complications were observed. To the best of our knowledge, this is the first study evaluating the role of HA treatment in patients with stable PD with perilesional administration of HA and without the use of local anesthesia.

### 4.2. Interpretation and Context

HA modulates the extracellular matrix’s composition, remodels the fibrotic tissue and enhances the elasticity of the penile connective tissue [7]. Moreover, high-molecular-weight HA is able to increase plaque hydration by improving lubrication and seems to have a role in tissue regeneration and repair [19]. Our results indicate that these physical effects can be mediated by Peyronie plaques from perilesional deposition, supporting the use of HA in stable-phase PD. In the past, there has been a substantial clinical gap in urological practice regarding the treatment of stable-phase PD [3]. Treatment options for individuals in the stable chronic phase have mainly been restricted to high-risk surgical adjustments or expensive intralesional collagenase injections, whereas the active, inflammatory phase is frequently targeted with various medicinal therapy [13]. Once the plaque has calcified or stabilized, traditional oral and topical medications frequently fail to produce significant effects, leaving a therapeutic gap for patients looking for less intrusive choices. Moreover, traditional methods often ignore the critical role of the surrounding tissue in favor of concentrating just on the thick core of the plaque. Our study addresses these clinical constraints by showing that the therapeutic advantages of HA can be transferred from perilesional deposition directly into the Peyronie’s plaques. By diffusing through the surrounding extracellular matrix, perilesional injection enables HA to improve tissue hydration, lessen mechanical stress, and possibly initiate localized remodeling of the stabilized collagen network. For patients with stable-phase PD, this mechanism offers a safe, convenient, and well-tolerated bridging therapy that bridges the gap between invasive surgical procedures and ineffectual conservative medicinal treatments.

### 4.3. Clinical Implications

The European Association of Urology summarizes that intralesional hyaluronic acid either alone or in combination with other treatments reduces pain and/or penile curvature in acute-phase PD but data are still limited [13]. Recently, Falcone et al. reported the clinical results of Perovial^®^ in a case series of 16 acute-phase PD patients [20]. With local anesthesia, they used a needle tunneling technique and distributed the vial across multiple injection sites. Regarding stable PD, only a single retrospective study by Cilio et al. provides findings in favor of HA injections. They also used local anesthesia in their study and report that injections were placed at the site of maximum curvature, but they do not specify the number of injections per procedure [11]. Achieving full intra-plaque infiltration of a volume of 2 mL is very challenging, while peri-plaque administration offers superior drug deposition and distribution. Our results in terms of penile curvature reduction and quality of life improvement are comparable to those from studies using intralesional injection [10,11,20]. Our statistical results must be compared to known minimal clinically important differences (MCID) in order to identify real-world patient utility when interpreting the effectiveness of perilesional HA injections. A reduction of at least 20% or an absolute decrease of at least 10° in penile curvature is frequently acknowledged as the threshold for a clinically noticeable benefit that directly affects the viability of sexual activity. In terms of erectile function, validated data for the standard IIEF-EF domain indicate that an increase of 2, 5, or 7 points represents a clinically significant change. On the other hand, we recognize that many psychometric instruments, such as the GAPD and some PDQ sub-scores, do not yet have defined MCID levels. However, a significant decrease in patient-reported symptom load and high post-treatment satisfaction are reflected in the overall favorable alterations in these scores. This means that a single perilesional injection of HA seems effective and can be delivered through one injection site without discomfort for patients and without local anesthesia. The observations in the present study are consistent with the findings in our previous feasibility study [8] but require confirmation through prospective trials.

### 4.4. Future Research

Recently, a systematic review and metanalysis on the use of HA on PD stated that the quality of studies was variable and that results are limited by scarcity of data. The effect of HA in stable PD seems promising, but the authors call for standardization of study regimens and randomized controlled trials [7]. We agree that more and better studies are needed and argue that future studies must be standardized regarding duration of symptoms before treatment, use of intra- and perilesional injections, number of injections and use of adjunct measures like vacuum devices. Later, we also need randomized studies with salt water as placebo.

### 4.5. Strengths and Limitations of This Study

A strength of this study is that it was conducted in a high-volume andrological center with standardized procedures by experienced urologists. There are several limitations. The study is a retrospective and exploratory analysis of a prospectively collected database, which precluded an a priori sample size calculation. It is non-randomized and there are no controls and no placebo. We recognized that the lack of placebo is considered as a major limitation of the study, but the comparison with a control group or placebo is out of the study aims. We did not register how often vacuum devices were used and it is difficult to assess the additional effect of this adjuvant measure. Eleven patients (19%) were excluded because of insufficient data. We acknowledge that missing data could theoretically correlate with a lack of treatment effect; however, the high homogeneity among baseline covariates strongly argues against the presence of a systematic selection bias that could skew our main findings. The curvatures were assessed by different methods at enrolment and follow-up, but we consider this to be a minor limitation only. However, the validity of our findings is strengthened by the implementation of a rigorous photographic protocol, which successfully minimized patient-dependent variance. Our study is, also, limited by the lack of objective quantification of patient adherence to the vacuum device protocol. While all included patients reported using the device at least three times per week, we could not analyze the independent effect of usage frequency or duration on outcomes. The 3-month follow-up period should be considered a limitation of the study. Most authors, however, believed that this time frame was adequate to assess the treatment’s effectiveness. Finally, we recognize that the lack of an HA-only or vacuum-only control group and the lack of objective digital monitoring for VED adherence are limitations of this study. Although research indicates that VED monotherapy is not very effective in reducing stable-phase plaque, our findings cannot be fully separated from its baseline synergy.

## 5. Conclusions

HA therapy in combination with vacuum devices seems to be able to significantly reduce penile curvature and improve patient-reported quality of life in patients with stable-phase Peyronie’s disease. Repeated perilesional Perovial^®^ injections, which can be administered through a single injection and deposited around the plaque without the need for ultrasound guidance or local anesthesia, seem a promising treatment in patients with stable-phase PD. Our study should be followed up with prospective comparative studies with better standardized protocols, including a control group.

## Figures and Tables

**Table 1:** Patient’s sociodemographic anamnestic, clinical characteristics at enrolment time.

No. of Analyzed Patients	47
Median age (median ± IQR)	58 (48–69)
Educational level	
Primary school	-
Secondary school	24 (51.0)
Post-secondary education	23 (49.0)
Sexually active (past month)	47 (100)
Current smoker	
No	30 (63.8)
Yes	17 (36.2)
BMI (kg/m^2^) (median ± IQR)	25 (23–31)
Charlson Comorbidities Index	
0–1	39 (82.9)
2	8 (17.1)
>2	-
Starting of symptoms (months) (median ± IQR)	15 (12–18)
Mean penile curvature (°) (median ± IQR)	45 (30–60)
Plaque size (millimeters) (median± IQR)	20 (15–31)
Incurvation side	
Dorsal	31 (65.8)
Right Lateral	0 (0)
Left Lateral	4 (8.6)
Right Lateral-Dorsal	12 (25.6)
Plaque position	
Proximal	8 (17.1)
Medium	8 (17.1)
Distal	31 (65.8)
IIEF-5 (median ± IQR)	20 (18–22)
PDQ (median ± IQR)	
Psychological and physical symptoms	12.6 (10.4–14.4)
Penile pain	6.4 (5.2–8.6)
Symptom bother	8.2 (7.0–9.3)

The table shows the patient characteristics at the enrolment. IQR: Interquartile range; BMI: Body Mass Index; IIEF-5: International Index of Erectile Function; PDQ: Peyronie’s Disease Questionnaire.

**Table 2:** Clinical, questionnaires and instrumental results at the follow-up evaluation.

No. of Patients Who Completed the Follow-Up		47	
	Before Treatment	After Treatment	Difference (*p*)
Mean penile curvature (°) (median ± IQR)	45 (30–60)	29 (19–43)	<0.001
Plaque size (millimeters) (median± IQR)	20 (15–31)	17 (11–26)	0.01
IIEF-5 (median ± IQR)	20 (18–22)	24 (24–25)	<0.001
PDQ (median ± IQR)			
Psychological and physical symptoms	12.6 (10.4–14.4)	9.8 (8.4–10.1)	<0.001
Penile pain	6.4 (5.2–8.6)	4.9 (4.0–7.1)	<0.001
Symptom bother	8.2 (7.0–9.3)	6.9 (6.0–7.4)	0.002

The table shows all clinical and questionnaires results at the 3-month follow-up visit. IQR: Interquartile range; IIEF-5: International Index of Erectile Function; PDQ: Peyronie’s Disease Questionnaire.

## Data Availability

The original contributions presented in this study are included in the article. For further inquiries, please contact the corresponding author.

## Abbreviations

The following abbreviations are used in this manuscript:

PD	Peyronie’s Disease
HA	Hyaluronic Acid
IIEF-5	International Index of Erectile Function-5
PDQ	Peyronie’s Disease Questionnaire
GAPD	Global Assessment of Peyronie’s Disease
